# Prediction models for intradialytic hypotension in hemodialysis patients: A protocol for systematic review and critical appraisal

**DOI:** 10.1371/journal.pone.0310191

**Published:** 2024-09-09

**Authors:** Zifeng Li, Luhuan Yang, Zuyang Xi, Wen Yi, Xiaoqian Zeng, Dongling Ma, Yunhong Lei

**Affiliations:** 1 Department of Traditional Chinese Medicine, The First College of Clinical Medical Science, Yichang Central People’s Hospital, Three Gorges University, Yichang City, Hubei Province, China; 2 Faculty of Medicine and Health Sciences, Universiti Putra Malaysia, Serdang, Selangor, Malaysia; 3 Yichang Hubo Medical Research Institute, Yichang City, Hubei Province, China; 4 Department of Nursing, the First College of Clinical Medical Science, Yichang Central People’s Hospital, Three Gorges University, Yichang City, Hubei Province, China; 5 Philippine Women’s University School of Nursing, Manila, Philippines; International University of Health and Welfare, School of Medicine, JAPAN

## Abstract

Intradialytic hypotension (IDH) is common in hemodialysis patients and can lead to several complications. Risk factors for IDH include demographic characteristics, comorbidities, dialysis procedure factors, and so on. Clinical studies on predictive models for dialysis-induced hypotension have shown inconsistent results. This systematic review aims to evaluate published prediction models for IDH, analyzing their characteristics, predictors, efficacy, and the methodological quality and applicability. The protocol has been prepared using the Preferred Items for Systematic Review and Meta-analysis Protocols (PRISMA-P) guidelines. The systematic review protocol for IDH prediction in hemodialysis patients has been registered with the International Platform of Registered Systematic Review and Meta-analysis Protocols (INPLASY2023110081, DOI: 10.37766/inplasy2023.11.0081). A comprehensive search across five major databases (PubMed, Web of Science, Cochrane Library, CNKI, and Wanfang) will be conducted for studies on prediction models of IDH among hemodialysis patients. Two researchers will independently screen literature, extract data, and evaluate the bias risk and applicability of included studies using prediction modelling study tools. This systematic review will provide critical insights into the efficacy and quality of reporting of the IDH model in hemodialysis patients. This will guide clinical staff in selecting the most appropriate IDH prediction model and inform future research endeavors in IDH prediction.

## Introduction

Chronic Kidney Disease (CKD) is characterized by persistent impairments in kidney structure or function lasting for at least three months, often arising from diverse causes [1]. Worldwide, the prevalence of CKD ranges between 11.7% and 15.1%, and this figure is on the rise. Typically, CKD is a progressive and irreversible condition that leads to a gradual decline in kidney function [2]. If not effectively managed, this decline can culminate in end-stage renal disease (ESRD). From 1990 to 2017, the mortality rates associated with CKD and ESRD saw a 41.5% increase [3]. The high prevalence, mortality rates, and medical costs associated with CKD and ESRD have made them a growing global public health concern.

Patients suffering from ESRD rely on renal replacement therapies (RRT) to sustain their lives, which include hemodialysis (HD), peritoneal dialysis (PD), and renal transplantation. Of these, HD is particularly vital, serving as the primary treatment for the majority of ESRD patients [1]. According to the 2017 United States Renal Data System (USRDS) report, the number of ESRD patients in the United States increased by 2.6% from 2016, reaching approximately 746,000, with over 63% undergoing HD treatment [4]. Additionally, Yang et al. estimated that by 2025, over 800,000 patients in China will require dialysis [5]. The HD functions like a semi-permeable membrane, draining blood to an external dialysis machine for exchange with a dialysate solution that mimics the body’s electrolyte composition. Employing ultrafiltration, diffusion, adsorption, and convection, this process effectively removes metabolic wastes and toxins while balancing water, electrolytes, and acid-base levels. The cleansed blood is then returned to the body [6].

While advancements in HD technology have improved patients’ quality of life, the prevalence of complications such as intradialytic hypotension (IDH) remains a concern. IDH, a common acute complication occurring during dialysis, has an incidence rate ranging from 7.5% to 50% [7, 8]. This condition presents significant risks, including arrhythmias, fistula thrombosis, fistula occlusion, and inadequate dialysis, and in severe cases, it can be life-threatening [9]. Therefore, early identification and intervention for IDH in HD patients are crucial. IDH is associated with various factors, including demographic characteristics (age, gender, race, height, weight, body mass index) [10–13], comorbidities (diabetes, cardiovascular diseases) [14–17], dialysis-related factors (interdialytic weight gain, ultrafiltration rate, dialysis duration, blood flow rate, dialysis modality) [18–22], and other factors (pre-dialysis blood pressure, serum phosphate levels, antihypertensive medication use) [23–26].

Despite numerous attempts to develop predictive models for IDH, the outcomes have remained inconsistent [27–29]. For instance, Bae TW et al. [27] proposed a model using a Multilayer Perceptron approach, which included six predictors and achieved a prediction accuracy of 81.5%. Similarly, Li Y et al. [28] developed a model based on an orthogonal learning mechanism with eight predictors, resulting in a prediction accuracy of 92.4%. Lin CJ et al. [29] utilized multiple linear regression, incorporating 15 predictors, and achieved an accuracy rate exceeding 80.0%. Discrepancies in predictive accuracy and the diverse methodologies used in model construction highlight inconsistencies across studies, especially in predictor selection, statistical methods, and model validation processes. Despite these advancements, there are currently no reliable risk assessment tools available for the early, personalized evaluation of hemodialysis (HD) patients.

To address these inconsistencies, our study protocol aims to comprehensively identify and evaluate existing predictive models for IDH. We will focus on assessing the statistical methods, sample sizes, predictor variables, and performance metrics of these models. The specific objectives of this review are to: 1) conduct a detailed comparison of existing predictive models for IDH; 2) summarize the key features of these models; 3) critically evaluate the methodologies employed in the included studies; 4)provide evidence-based recommendations for future model development.

## Methods and analysis

This systematic review protocol was registered with the International Platform of Registered Systematic Review and Meta-analysis Protocols (INPLASY) on November 20, 2023(https://inplasy.com/inplasy-2023-11-0081/). It follows the Preferred Reporting Items for Systematic Reviews and Meta-Analyses Protocols (PRISMA-P) guidelines [30] (S1 Appendix). The protocol employs a thorough methodology, using the Critical Appraisal and Data Extraction for Systematic Reviews of Prediction Modeling Studies (CHARMS) checklist to deeply evaluate and gather data for prediction modeling studies [31] (S2 Appendix). Bias risk in the reviewed prediction models is assessed using the Prediction Model Risk of Bias Assessment Tool (PROBAST) tool [32] (S3 Appendix). Furthermore, this protocol is reported reporting as per the Transparent Reporting of a multivariable prediction model for Individual Prognosis Or Diagnosis (TRIPOD) statement for multivariable prediction models [33] (S4 Appendix).

### Eligibility criteria

Table 1 outlines the eligibility criteria for the studies, guided by the PICOTS (population, index, comparator, outcome, timing, setting) framework [34]. an evolved version of the classic PICO framework, incorporating considerations of time and clinical setting. Selection of studies will be based on the eligibility criteria.

**Table 1:** Eligibility criteria for the systematic review framed according to the PICOTS system.

Item	Definition
*Population*	*Hemodialysis patients aged≥18 years old*
*Index*	*Any diagnostic prediction model to predict the risk of IDH in HD patients*
*Comparator*	*Not applicable*
*Outcome*	*IDH*
*Timing*	*Outcome occurred during HD*
*Setting*	*Settings including wards*, *intensive care units*, *and dialysis treatment rooms where hemodialysis can be conducted*.

### Population

Studies reporting on prediction models for IDH in HD patients aged≥18 years old will be considered for inclusion.

### Intervention

Diagnostic prediction modeling studies, whether validated or not, will be considered for inclusion in the systematic review if they aim to aid clinicians in diagnosing IDH.

### Outcome

The focus of this study is IDH, for which there is no global consensus on definition. We adopt the definitions provided by the National Kidney Foundation (NKF) in the Kidney Disease Outcome Quality Initiative (K/DOQI) guidelines. Specifically, IDH is characterized by a systolic blood pressure drop of 20mmHg or a mean arterial pressure decrease of 10mmHg during dialysis, along with symptoms like abdominal discomfort, yawning, nausea, vomiting, and muscle cramps [35]. Considering the variation in standards for IDH prediction models, our review will not strictly limit the definition of IDH. All related studies will be included, and the reference definitions for IDH will be detailed in the tables.

### Timing

Only studies focusing on diagnostic prediction models for hypotension during dialysis will be included. Models predicting hypotension before or after dialysis will be excluded.

### Setting

Diagnostic prediction models intended for clinical use across various dialysis settings, including dialysis centers, intensive care units, and general wards, will be considered for inclusion in the review.

### Types of studies and limits

This review does not limit study designs and encompasses all research on prediction models for IDH, except for those involving animal studies. The literature published up to December 31, 2023, will be reviewed.

### Search methods for identification of studies

#### Information sources

We will search the following five databases: PubMed, Web of Science, Cochrane Library, CNKI, and Wanfang. Additionally, manual review of references in the selected literature will be conducted to identify potentially relevant studies.

#### Search strategy

This study will employ a three-step search strategy to ensure comprehensive literature coverage. Initially, two experienced researchers will develop the search strategy. The search terms include those related to hemodialysis/dialysis, predictive modeling, and hypotension (see S5 Appendix), and a preliminary search will be performed on PubMed and CNKI. To further refine the search terms, an expert from the dialysis unit will be consulted. Subsequently, the searches will be conducted by two independent researchers. In the final phase, a detailed review of the selected literature and related references will be performed, to find additional studies meeting the inclusion criteria.

### Data collection and analysis

#### Data management

All bibliographic records will be imported into Endnote X9, a reference management software. The software’s built-in functions will be used to identify and remove duplicate entries.

#### Selection process

**Fig 1:**
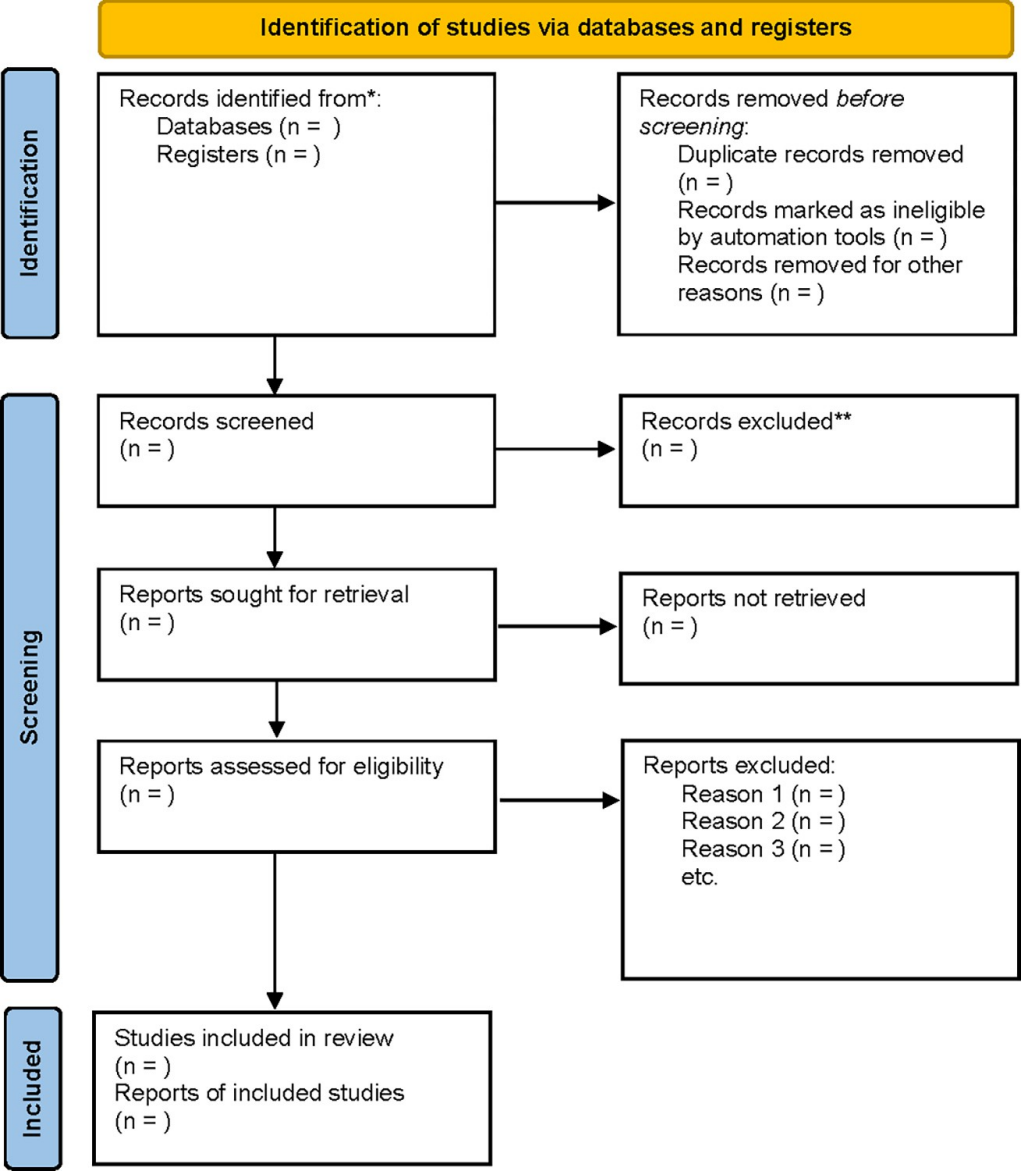
Flow chart of study selection.

The two researchers conducted the screening independently. Initially, both researchers independently reviewed titles and abstracts to determine study relevance. Studies with uncertain or potential relevance, including conference papers without abstracts, will be retained for further review. Subsequently, both researchers will independently read the screened full texts of the studies and rigorously evaluate it against the predefined eligible criteria. When required, conflicts will be addressed by consulting a third researcher. This process will follow the PRISMA diagram (See Fig 1).

#### Data extraction

Two independent reviewers will conduct data extraction from selected studies using a standardized electronic Excel spreadsheet. The Excel sheets, organized according to the CHARMS checklist, cover 11 domains such as target information, data sources, participants, predicted outcomes, candidate predictors, sample size, and missing data [31]. The evaluation of model performance metrics should encompass not only discrimination and calibration, but also the outcomes derived from decision curve analysis and net benefit assessments [36, 37]. Finally, the researchers will cross-check the extracted data and review the full text to identify and correct discrepancies, ensuring accuracy.

#### Critical appraisal

The PROBAST tool will be employed for a critical assessment of bias risk and applicability within the selected risk prediction modeling studies [32]. This assessment will encompass four primary domains: the study population, predictor variables, outcomes, and the statistical analyses, with a total of 20 items. Two independent researchers will conduct the assessments, and any discrepancies will be resolved by a third researcher’s decisive judgment. Furthermore, a detailed tabulation will display the findings from the bias risk and applicability assessments.

#### Qualitative data synthesis of prediction models

Data from included studies on prediction models will be systematically tabulated to facilitate the comparison of predicted survival outcomes, final model predictors, and associated performance metrics [31]. When available, uncertainty measures for these metrics will be reported or estimated through established methods [34].

#### Reporting and presentation of findings

Systematic review will be reported in accordance with the Preferred Reporting Items for Systematic Reviews and Meta-Analyses (PRISMA) guidelines [38] and the TRIPOD statement [33] to improve report transparency and completeness.

## Discussion and conclusion

Despite numerous reviews focusing on hypotension in dialysis patients, these primarily discuss risk factors and intervention strategies [20, 39–41]. An initial review reveals studies on predictive models for IDH, yet the findings are inconsistent [27–29]. This systematic review will offer a detailed overview of all studies on diagnostic predictive models for IDH in hemodialysis patients. To our knowledge, this is the first systematic review dedicated to prediction models for IDH in hemodialysis patients. This review will equip clinicians with essential insights to choose an optimal prediction model for enhanced hypotension management during hemodialysis, by evaluating the characteristics, predictive factors, and performance metrics.

### Strengths and limitations

This study will be conducted in accordance with the guidelines of the Cochrane Handbook and aims to provide high-quality evidence on prediction models for IDH. By describing in detail the basic characteristics, predictors, and predictive performance of different prediction models, this study will help clinical staff to select appropriate prediction models to reduce the risk of IDH in hemodialysis patients. The systematic review of prediction models not only facilitates the selection of the optimal model but also clarifies its applicability and generalizability, establishing a basis for further evaluation and validation [34, 42].

However, the lack of recognized diagnostic criteria for IDH presents challenges in accurately assessing incidence, integrating data, and developing evidence-based prevention and treatment guidelines. This systematic review primarily conducted qualitative descriptive analyses without merging model parameters. Variations in clinical backgrounds, patient baseline characteristics, and statistical methodologies across the studies likely contributed to increased heterogeneity. Overall, this study will offer a thorough assessment of models published for IDH prediction.

## Supporting information

S1 Appendix Preferred Reporting Items for Systematic Review and Meta -Analysis Protocols (PRISMA-P). (PDF)

S2 Appendix Critical appraisal and data extraction for systematic reviews of prediction modeling studies (CHARMS). (PDF)

S3 Appendix Prediction Model Risk of Bias Assessment Tool (PROBAST). (PDF)

S4 Appendix Transparent Reporting of a multivariable prediction model for Individual Prognosis Or Diagnosis (TRIPOD). (PDF)

S5 Appendix Search strategy. (PDF)
