# Supplementary material for: Prediction models for intradialytic hypotension in hemodialysis patients: A protocol for systematic review and critical appraisal
Source: PLoS One. 2024 Sep 9;19(9):e0310191. doi: 10.1371/journal.pone.0310191 (PMC11383225; doi:10.1371/journal.pone.0310191)
Supplement: S5 Appendix — (PDF) [file pone.0310191.s005.pdf]

Table 2. Full search string for the database "Pubmed"

Pubmed Search Strategy		
1. Population	*Subject Headings*	*"Hemodialysis"[MeSH Terms] OR*<br>*"Dialysis"[MeSH Terms] OR*<br>*"Renal Dialysis"[MeSH Terms]*
	Keywords	*"Dialyses"[Title/Abstract] OR*<br>*"Dialysis Extracorporeal"[Title/Abstract] OR*<br>*"Dialysis Renal"[Title/Abstract] OR*<br>*"Extracorporeal Dialysis"[Title/Abstract] OR*<br>*"Dialyses Extracorporeal"[Title/Abstract] OR*<br>*"Dialyses Renal"[Title/Abstract] OR*<br>*"Extracorporeal Dialyses"[Title/Abstract] OR*<br>*"Hemodialyses"[Title/Abstract] OR*<br>*"Renal Dialyses"[Title/Abstract]*
2. index prediction model	***AND***	
	Subject Headings	
	Keywords	*"risk prediction"[Title/Abstract] OR*<br>*"model"[Title/Abstract] OR*<br>*"Predict"[Title/Abstract] OR*<br>*"prediction model"[Title/Abstract] OR*<br>*"nomogramwos"[Title/Abstract] OR*<br>*"estimate"[Title/Abstract] OR*<br>*"area under the curve"[Title/Abstract] OR*
3. Outcome	***AND***	
	Subject Headings	*"Hypotension"[MeSH Terms]*
	Keywords	*"Blood Pressure Low"[Title/Abstract] OR*<br>*"Hypotension Vascular"[Title/Abstract] OR*<br>*"Low Blood Pressure"[Title/Abstract] OR*<br>*"Vascular Hypotension"[Title/Abstract] OR*<br>*"Intradialytic Hypotension"[Title/Abstract]*
